# Outcomes of para-sacral transcutaneous electric nerve stimulation in treatment of primary and refractory overactive bladder among children

**DOI:** 10.1007/s11255-024-04006-8

**Published:** 2024-05-13

**Authors:** Moetaz Dahshan Gomaa, Hany Aref AbdAllah, Iman Yehia Ismail, Isaac Samir Wasfy, Mahmoud Hegazy Sherief

**Affiliations:** Department of Urology, Faculty of Medicine, Suez Canal University, Ismailia, 41511 Egypt

**Keywords:** Overactive bladder, Children, Refractory, Parasacral transcutaneous electric nerve stimulation

## Abstract

**Background:**

Overactive bladder (OAB) is the most prevalent lower urinary tract dysfunction among children. Refractory OAB lacks response to the first line treatments, including anticholinergic, and it is a major clinical challenge. This study aims to compare the efficacy of para-sacral transcutaneous electric nerve stimulation (PS-TENS) in the treatment of primary and refractory OAB among children.

**Methods:**

A prospective, randomized clinical study included 64 children aged 5–12 years with OAB divided equally into two groups: group (1) included 32 children with treatment-naive OAB, while group (2) included 32 children with refractory OAB who failed complete course of anticholinergics. Both groups received PS-TENS three times weekly for 12 weeks. Detailed medical history, voiding diary, ultrasound, and uroflowmetry with EMG were performed at baseline and after 12 weeks of treatment. Filling cystometry was performed for children with refractory OAB before and after PS-TENS.

**Results:**

After the end of treatment the number of children with urge incontinence decreased significantly among the two groups: from 25 to 13 and 27 to 15 for the primary and refractory groups, respectively. Micturition frequency per 24 h decreased significantly among children with primary OAB. Bladder capacity increased significantly and the resting detrusor pressure decreased significantly among children with refractory OAB.

**Conclusions:**

PS-TENS is an effective and safe treatment option for children with OAB. The magnitude of improvement is higher among children with primary as compared to refractory OAB.

## Background

Overactive bladder (OAB) is the most common lower urinary tract dysfunction among children [1], and it is documented in about 6% of girls and 3.8% of boys at age 7 years [2]. OAB has significant complications, such as pelvic floor dysfunction, recurrent urinary tract infection (UTI), vesicoureteral reflux (VUR) and various forms of urinary incontinence. In addition, OAB is related to negative quality-of-life issues among the affected children, such as low self-esteem, social isolation, shyness, aggression and transgression. Besides, untreated OAB in childhood increases the risk of severe and refractory symptoms in the adult life [3].

Overactive bladder in children is defined by the International Children's Continence Society (ICCS) as the presence of urinary urgency that is usually accompanied by urge incontinence, frequency and/or nocturia. It is a clinical diagnosis based on symptoms only without the need of any additional signs or urodynamic findings to fulfill its diagnosis [4–6].

Anticholinergics represent the mainstay of medical treatment for OAB in children [7]; these agents relax detrusor smooth muscles thus increase bladder capacity, decrease the threshold and strength of bladder contractions and consequently delay the initial desire to void [8]. Solifenacin succinate has longer half-life, better bioavailability, potentially fewer side effects and better tolerance than oxybutynin owing to its selectivity to M3 muscarinic receptors in the urinary bladder [9]. That is why it has been used off-label for the treatment of voiding dysfunction in children. Recently, phase III randomized placebo-controlled trials have proven the effectiveness and tolerability of solifenacin in children and it recently gained FDA approval for the use in neurogenic OAB in pediatric patients as young as 2 years of age [7, 10, 11].

Several drawbacks make anticholinergics far from being an ideal treatment for OAB in children: first, intolerable side effects such as constipation, dry mouth, tachycardia, cognitive impairments, hyperthermia, and headache can result in early treatment discontinuation. Second, about 30–40% of children with OAB do no respond to anticholinergics, this case is usually referred to as refractory OAB [12]. Finally, despite being quite effective in alleviating symptoms of OAB, anticholinergics are imperfect for the treatment of dysfunctional voiding, since they modulate bladder storage not voiding [13].

Transcutaneous electrical nerve stimulation (TENS) was the first type of neuromodulation described in the pediatric population, and it was introduced as an alternative treatment for children with OAB refractory to anticholinergics [14–16].

## Methods

### Study setting and participants

**Fig. 1:**
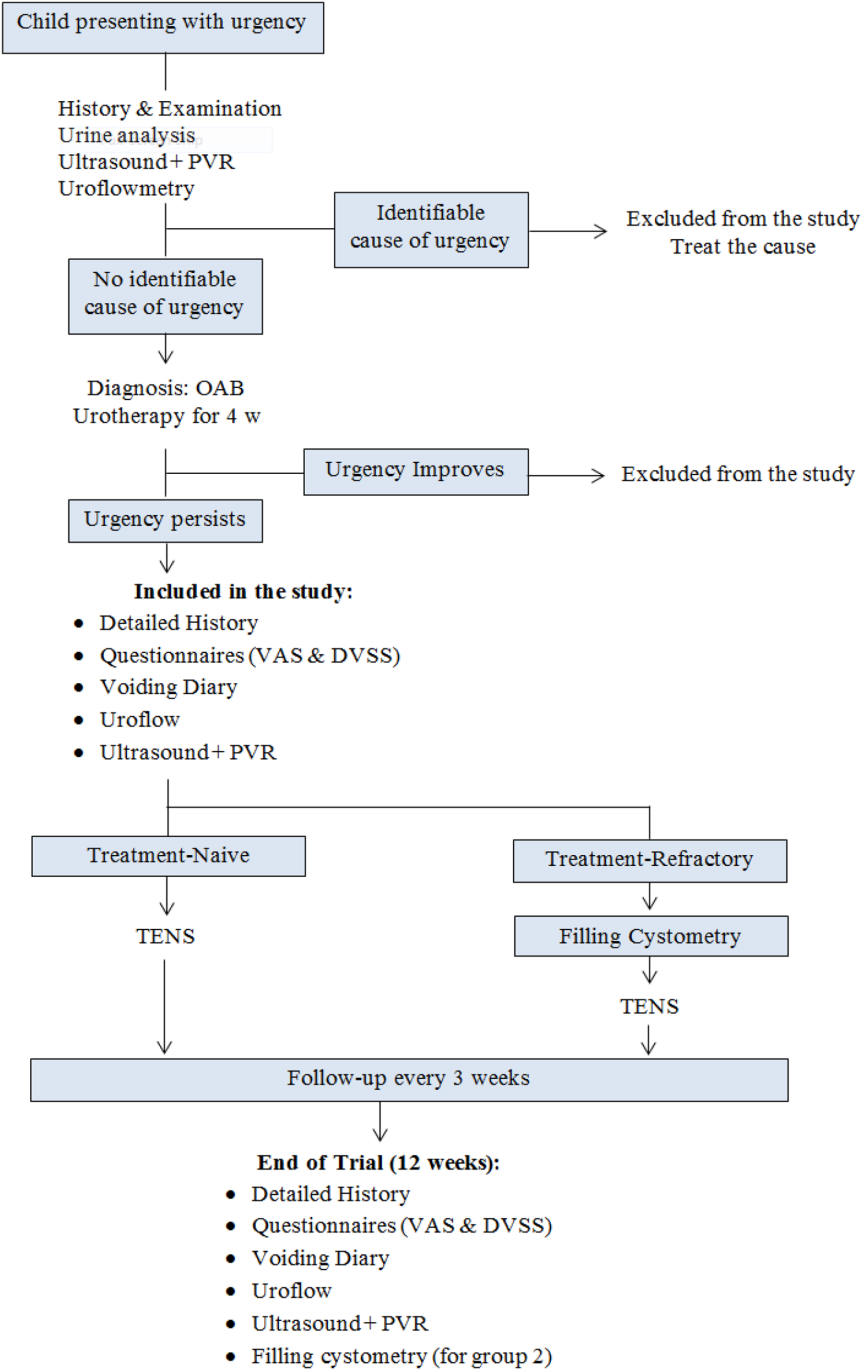
CONSORT chart of the study

The current study is a prospective clinical trial performed in the department of urology Suez Canal University from 6/2017 till 3/2020. It enrolled 64 children with OAB who either did not receive any prior treatment (Group 1: treatment-naive), or who had persistent OAB symptoms despite complete course of anticholinergics (Group 2: refractory OAB). The study was approved by the Scientific Research Ethics Committee of Suez Canal University, Egypt. This clinical trial was reported following the Consolidated Standards of Reporting Trials (CONSORT) guidelines (Fig. 1).

### Inclusion and exclusion criteria

We included male and female children aged 5–12 years, diagnosed with OAB according to the criteria of the International Children's Continence Society [6]: persistent voiding urgency with or without urgency urinary incontinence, usually associated with urinary frequency and nocturia. An additional inclusion criterion for group (2) was lack of response to anticholinergics taken for at least 1 month with compliance rate ≥ 80%. We excluded children with other possible causes of urgency, such as urinary tract infection, bladder stones, vesicoureteral reflux, neurogenic bladder and infra-vesical obstruction.

### Sample size calculation

The sample size was calculated using MedCalc software version 17.9, with a power of 80%, confidence level of 95%, *α* = 0.05 and *β* = 0.2; the number of study sample was 29 plus 10% drop-out rate = 32 patient per group.

### Study plan

Children with symptoms suggesting OAB were evaluated by a complete medical history, physical examination, 3-day voiding diary, urine analysis, uroflowmetry and pelvi-abdominal ultrasound. Children with no evident pathology that can be responsible for their symptoms were diagnosed with OAB; initial conservative treatment (i.e., urotherapy) was tried for all children for maximum duration of 4 weeks. Urotherapy consisted of education of the child and his parents about OAB and about normal LUT function, regular voiding at intervals of about 3 h with minimum of seven voids per day, avoiding caffeine, soda, chocolate, citrus fruits and other bladder irritants, urinating before bedtime, ingesting large amounts of fluids throughout the day while reducing fluid intake during night, using toilet seat reducer and/or foot rest when necessary [17].

**Fig. 2:**
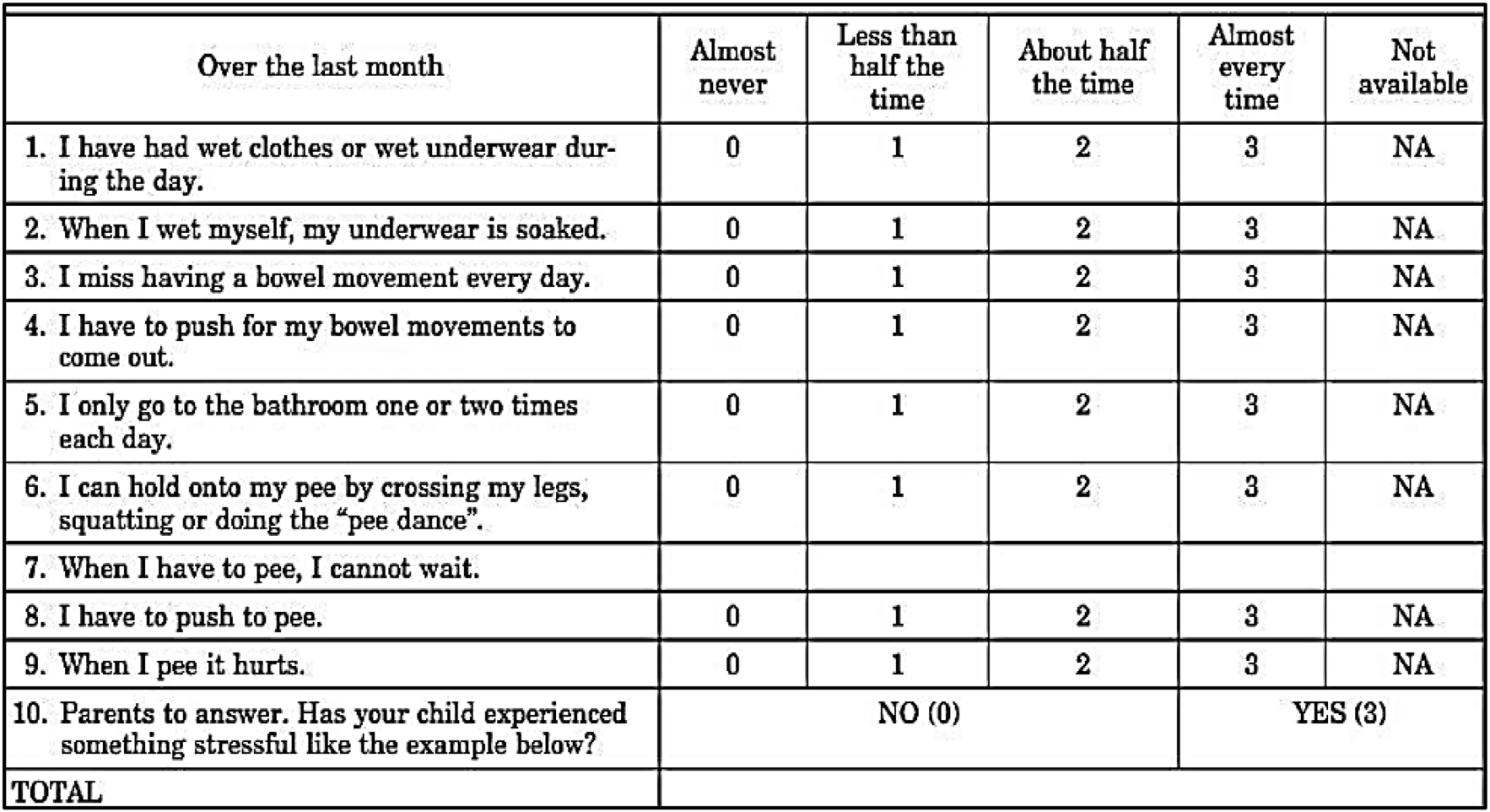
Dysfunctional Voiding Symptom Score (DVSS)

**Fig. 3:**
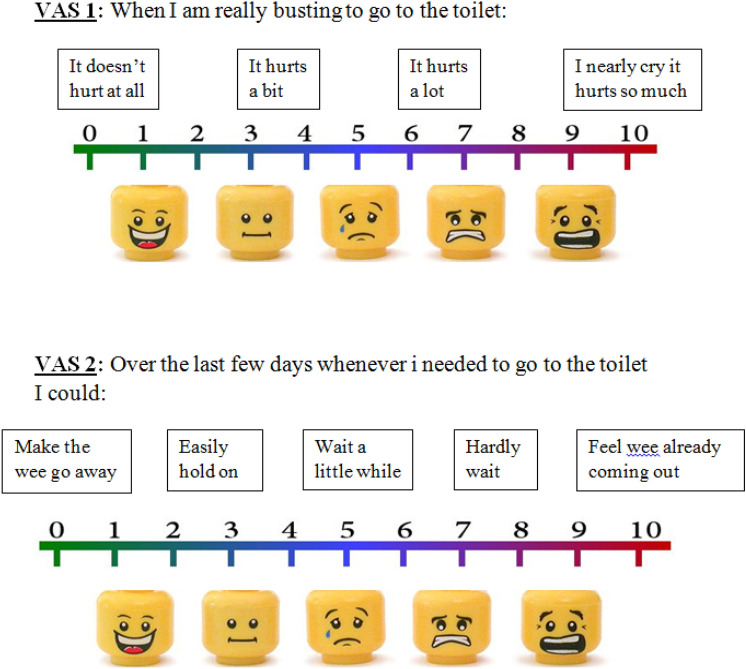
Visual Analog Scale (VAS)

Children who responded to urotherapy were excluded from this study, while those who did not respond were enrolled into the study after explaining the benefits and risks to the parents and obtaining a written consent. Quantification of symptoms was done using Dysfunctional Voiding Symptom Score (DVSS) which is designed by Farhat et al. [18] (Fig. 2) and Visual Analog Scale (VAS) (Fig. 3). Children in group (1) started PS-TENS directly, while children in group (2) had filling cystometry done before starting PS-TENS.

### Electric stimulation

**Fig. 4:**
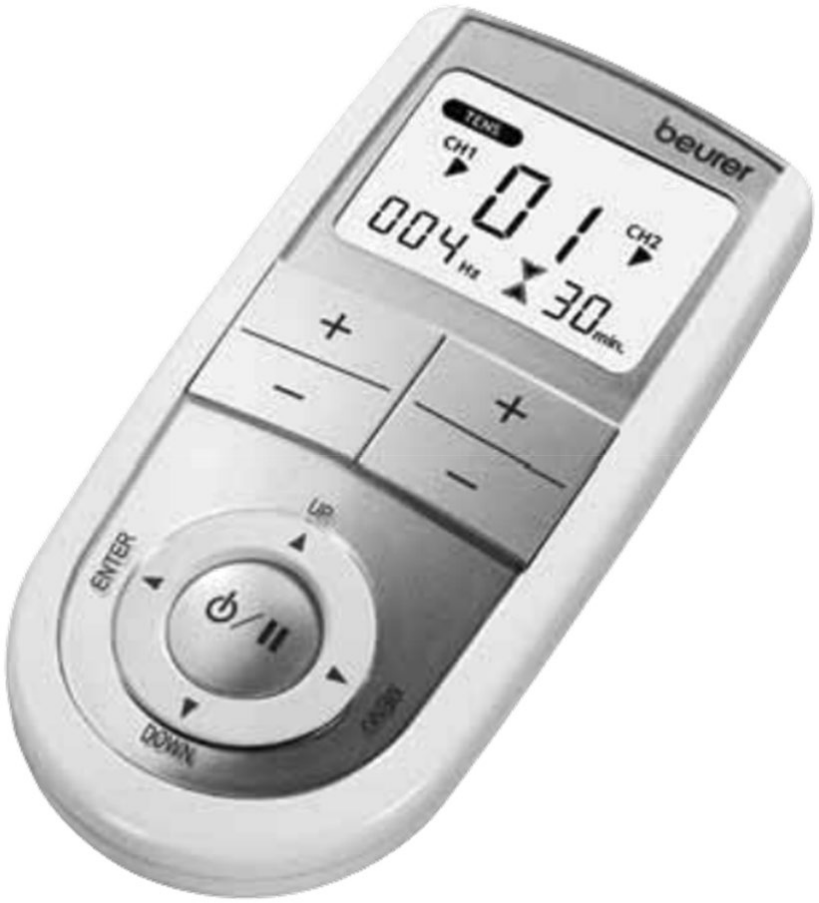
TENS device

**Fig. 5:**
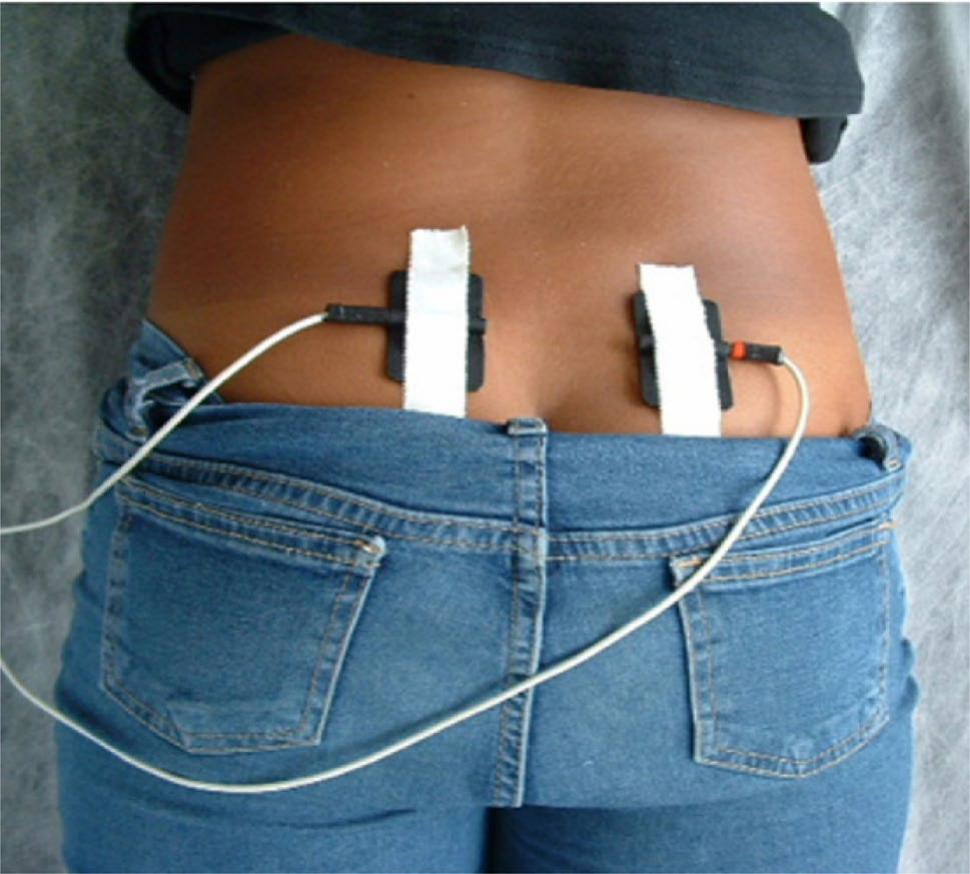
Location of the skin patches

Children in both groups received parasacral transcutaneous electric nerve stimulation at the office using the EM 41 electric stimulation device (Beurer GmbH^®^, Ulm Germany) (Fig. 4). Two superficial 3.5 cm gel electrodes (i.e., skin patches) were placed symmetrically on each side of S3 sacral foramen (Fig. 5) The surface anatomy of S3 nerve root at their exit from the sacral foramina is one finger breadth lateral to the midline spinous process and two finger breadth below the level of posterior superior iliac spine [14, 19]**.** A symmetrical biphasic square current pulse was used, with a frequency of 10 Hz, pulse width 700 ms and intensity increased gradually according to child's tolerance up to the level just below the motor threshold. Each session lasted for 20 min, three times weekly for 12 weeks [20].

### Study end-point

After completing the course of PS-TENS (12 weeks), all efficacy and safety parameters were repeated: DVSS, VAS, 2-day voiding diary, pelvi-abdominal ultrasound and uroflowmetry with EMG lag time. Filling cystometry was repeated for children with refractory OAB.

## Results

All children in the study were comparable regarding their sociodemographic characteristics, with no statistically significant differences between age, gender, residence and body mass index. The mean age was 7.9 ± 1.4 years for group (1) and 8.8 ± 1.6 years for group (2), 63% of group (1) are males versus 66% of group (2).

Table 1 demonstrates improvement of symptoms after treatment, the number of children with daytime incontinence and nocturnal enuresis was significantly reduced among the two groups, with group (1) showing significantly higher number of children with improvement of daytime symptoms. All children in the study had statistically significant reduction in the visual analog scale and dysfunctional voiding symptom score.

**Table 1:** Improvement of symptoms after treatment

Variable		TENS	Refractory	*p* value
No. of children with Incontinence	Pre	25 (78%)	27 (84%)	0.45
	Post	13 (41%)	15 (47%)	0.04
	*p* value	0.03	0.04	
No. of children with nocturnal enuresis	Pre	25 (78%)	28 (88%)	0.28
	Post	8 (25%)	10 (31%)	0.07
	*p* value	0.001	0.01	
VAS (Mean ± SD)	Pre	6.9 ± 2	8.3 ± 1.5	0.51
	Post	5.1 ± 2.6	7.6 ± 1.6	0.12
	*p* value	0.01	0.04	
DVSS (Mean ± SD)	Pre	19.8 ± 5.9	17.7 ± 7.1	0.25
	Post	16.9 ± 1.2	7.3 ± 8.1	0.01
	*p* value	0.04	0.08	

Table 2 demonstrates the changes of voiding diary parameters after treatment, all children in the study demonstrated highly significant improvement of all voiding diary parameters. The magnitude of improvement was significantly higher among children with treatment-naïve OAB as compared to those with treatment-refractory OAB.

**Table 2:** Changes of voiding diary after treatment

Variable		TENS	Refractory	*p* value
Micturition frequency/24 h	Pre	9.2 ± 3.1	10.3 ± 1.9	0.16
	Post	7 ± 1.9	9.5 ± 3.8	0.001
	*p* value	0.003	0.19	
Mean voided volume (ml)	Pre	103.7 ± 1.3	82.6 ± 8.3	0.001
	Post	111.8 ± 15.3	85.3 ± 9.1	0.001
	*p* value	0.02	0.3	
Daytime max. voided vol. (ml)	Pre	123.7 ± 7.3	92.8 + 7.7	0.001
	Post	130.8 ± 15.3	99 + 15.5	0.001
	*p* value	0.05	0.04	
Urgency episodes /24 h	Pre	4.6 ± 1.4	7.3 ± 1.6	0.001
	Post	3 ± 3.2	5.8 ± 2.8	0.002
	*p* value	0.01	0.01	
Incontinence episodes/24 h	Pre	4.1 ± 2.6	6 ± 2.3	0.006
	Post	2.7 ± 2.8	4.9 ± 2.9	0.008
	*p* value	0.04	0.02	
Incontinence-free days/week	Pre	3	4	0.58
	Post	5	6	0.11
	*p* value	0.04	0.03	
Incontinence-free nights/week	Pre	2	3	0.72
	Post	5	5	0.91
	*p* value	0.02	0.03	

Table 3 demonstrates the improvement of cystometry parameters after PS-TENS among children with refractory OAB, improvement was statistically significant for all parameters except for the number of children with cystometry-proven detrusor overactivity (DO), and the number of involuntary detrusor contractions (IDCs) in each cystometry.

**Table 3:** Changes of cystometry parameters after PS-TENS among children with refractory OAB

Variable	Before TENS	After TENS	*p* value
No. with detrusor overactivity	30 (94%)	24 (75%)	0.08
Resting detrusor pressure (mm H2O) (mean ± SD)	14.6 ± 3.6	11.5 ± 4.6	0.001
No. of involuntary detrusor contractions (mean ± SD)	4.4 ± 1.4	3.5 ± 2.4	0.06
Maximum involuntary detrusor contractions (mm H2O) (mean ± SD)	14.6 ± 3.6	11.5 ± 4.6	0.001
Cystometric bladder capacity (ml) (mean ± SD)	91 ± 15.8	108 ± 20.1	0.03

## Discussion

All studied children were comparable regarding their initial sociodemographic data at recruitment into the study, there was no statistically significant difference regarding age, gender, residence and BMI. This reduces the possibility of selection bias, and ameliorates possible unknown confounding factors. The mean age at presentation was around 8 years for the two groups, which is the age of entering primary school, and children of the refractory OAB group had slightly higher mean age, probably due to the time of trying several treatment lines before being diagnosed with refractory OAB.

The number of children in this study who complained of urge incontinence was significantly reduced among the two groups: from 25 to 13 (*p* = 0.001) and 27 to 15 (*p* = 0.04) for the primary and refractory groups, respectively. The rates of complete response were higher for NE than daytime incontinence; this may reflect different pathophysiologic mechanisms underlying urge incontinence and nocturnal enuresis.

Comparable results were reported from a prospective study of children with OAB performed by [21] who included 83 children with a mean age of 7.8 ± 2.8 years, diagnosed with OAB, and gave them PS-TENS twice weekly for a total of 20 sessions each lasted for 20 min. They reported complete resolution of symptoms in 56.6% of children.

In the present study we assessed urgency severity by VAS which was applied in two questions, and then the mean score was calculated. All children and/or parents in our study reported significant improvement of symptom severity as objectively assessed by the VAS, which reduced after treatment from 6.9 ± 2.1 to 4.8 ± 2.6 (*p* = 0.005), and 8.3 ± 1.5–7.6 ± 1.6 (*p* = 0.04) for groups 1 and 2, respectively. Similarly, we found significant reduction of the mean DVSS among the two groups: from 19.8 ± 5.1 to 6 ± 3.9 (p = 0.04), and 17.7 ± 7.1–7.3 ± 8.1 (*p* = 0.08) for the primary and refractory groups, respectively. The discrepancy between VAS and DVSS can be explained by the subjective nature of the VAS, which encouraged the parents to emphasize the severity of the disease and their bother by its resistance to treatment.

Different results were reported by [22] who reported no difference in symptomatic improvement among children and adolescents with OAB who received urotherapy plus PS-TENS and those who received urotherapy plus sham electric stimulation. These findings may be related to the small sample size (20 patients per group) and the heterogeneity of the sample which included children and adolescents.

In the current study all children had statistically significant improvement of all voiding diary parameters. Among children in the TENS group micturition frequency per 24 h was reduced from 9.2 ± 3.1 to 7.1 ± 1.9 (*p* = 0.003), mean voided volume increased from 103.7 ± 7.3 to 111.8 ± 15.3 (*p* = 0.02), maximum voided volume per day increased from 109.5 ± 4.6 to 115.6 ± 13.8 ml (*p* = 0.03), mean number of urgency episodes per day decreased from 4.6 ± 1.4 to 3 ± 3.2 (*p* = 0.01), and the mean number of urge incontinence episodes per day decreased from 4.1 ± 2.6 to 2.7 ± 2.8 (*p* = 0.04). In a prospective study by [23], they randomly allocated children with OAB into either active treatment with PS-TENS or sham electric stimulation into the scapular region, they reported statistically significant improvement of the average and maximum voided volumes (*p* = 0.003 and 0.001, respectively) as well as the number of voids per day (*p* = 0.01).

In the present study, we performed filling cystometry for children with refractory OAB before and after the course of PS-TENS, we found highly significant reduction of resting detrusor pressure from 14.6 ± 3.6 to 11.5 ± 4.6 cm H_2_O (*p* = 0.001) after PS-TENS. Maximum amplitude of involuntary detrusor contractions decreased from 14.6 ± 3.6 to 11.5 ± 4.6 cm H_2_O (*p* = 0.001) and cystometric bladder capacity increased from 91 ± 15.8 to 108 ± 20.1 ml (*p* = 0.03), while the reduction of number of involuntary detrusor contractions was not statistically significant: from 4.4 ± 1.4 to 3.5 ± 2.4 (*p* = 0.06).

The only study reporting the urodynamic outcomes of PS-TENS among children with OAB was performed by [20]. They included 18 children with idiopathic OAB, who undergone cystometry before starting PS-TENS in 20 sessions each lasting for 20 min. Twelve of the children performed follow-up cystometry after completing TENS. Bladder capacity improved significantly after TENS while there was no significant reduction neither in the average number of inhibited contractions (*p* = 0.56) nor in the highest detrusor pressure during detrusor contraction (*p* = 0.2).

Children participating in the aforementioned study had significant symptomatic improvement despite non-statistically significant improvement of urodynamic parameters. The authors hypothesize that TENS induced neuroplasticity that improved symptoms and bladder capacity, but did not alter the brains capacity to inhibit IDCs. An alternative theory is that TENS modified cerebral interpretation of IDCs thus abolishing symptoms without disappearance of the detrusor contractions. A final theory is that filling cystometry is a non-physiological situation that may induce asymptomatic IDCs which are clinically insignificant [20].

## Conclusion

We conclude that PS-TENS is an effective and safe treatment option for children with OAB. Parasacral TENS is a valuable option for children who failed medical treatment with modest symptomatic improvement but significant reduction of detrusor pressure, thus protecting the upper urinary tract.

## Data Availability

All research data and materials are available with the corresponding author.

## Abbreviations

DO: Detrusor overactivity
DVSS: Dysfunctional voiding symptom score
No.: Number
OAB: Overactive bladder
IDCs: Involuntary detrusor contractions
Pdet: Detrusor pressure
PS-TENS: Para-sacral transcutaneous electric nerve stimulation
SD: Standard deviation
TENS: Transcutaneous electric nerve stimulation
VAS: Visual analog scale

## References

[1] Franco I (2007) Overactive bladder in children. Part 1: pathophysiology. J Urol 178(3):761–768 17631323 10.1016/j.juro.2007.05.014

[2] Franco I (2016) Overactive bladder in children. Nat Rev Urol 13(9):520–532 27530266 10.1038/nrurol.2016.152

[3] Tekgul S, Nijman R, Hoebeke P, Canning D, Bower W, von Gontard A. Diagnosis and management of urinary incontinence in childhood. https://www.ics.org/Publications/ICI_4/files-book/Comite-9.pdf

[4] Abrams P, Cardozo L, Fall M et al (2003) The standardization of terminology in lower urinary tract. Urology 61(1):37–49 12559262 10.1016/S0090-4295(02)02243-4

[5] Nevéus T, von Gontard A, Hoebeke P et al (2006) The standardization of terminology of lower urinary tract function in children and adolescents: report from the standardisation committee of the international children’s continence society. J Urol 176(1):314–324 16753432 10.1016/S0022-5347(06)00305-3

[6] Austin P, Bauer S, Bower W et al (2016) The standardization of terminology of lower urinary tract function in children and adolescent. Neurourol Urodyn 35(4):471–481 25772695 10.1002/nau.22751

[7] Nadeau G, Schroder A, Moore K et al (2014) Long-term use of solifenacin in pediatric patients with overactive bladder: extension of a prospective open-label study. Can Urol Assoc J 8(3–4):118–123 24839481 10.5489/cuaj.1356 PMC4001633

[8] Franco I (2012) Functional bladder problems in children: pathophysiology, diagnosis, and treatment. Pediatr Clin North Am 59(4):783–817 22857829 10.1016/j.pcl.2012.05.007

[9] Kreder KJ (2006) Solifenacin. Urol Clin North Am 33(4):483–490 17011384 10.1016/j.ucl.2006.06.008

[10] Bolduc S, Moore K, Nadeau G et al (2010) Prospective open label study of solifenacin for overactive bladder in children. J Urol 184(4):1668–1673 20728124 10.1016/j.juro.2010.03.124

[11] Newgreen D, Bosman B, Hollestein-Havelaar A et al (2017) Solifenacin in children and adolescents with overactive bladder: results of a phase 3 randomised clinical trial. Eur Urol 71(3):483–490 27687820 10.1016/j.eururo.2016.08.061

[12] Park S, Pai K, Kim J et al (2014) Efficacy and tolerability of anticholinergics in Korean children with overactive bladder: a multicenter retrospective study. J Korean Med Sci 29(11):1550 25408588 10.3346/jkms.2014.29.11.1550 PMC4234924

[13] Arlen A (2017) Dysfunctional voiders—medication versus urotherapy? Curr Urol Rep 18(2):14 28213858 10.1007/s11934-017-0656-0

[14] Hoebeke P, Laecke E, Everaert K et al (2001) Transcutaneous neuromodulation for the urge syndrome in children: a pilot study. J Urol 166(6):2416–2419 11696801 10.1016/S0022-5347(05)65605-4

[15] Malm-Buatsi E, Nepple K, Boyt M et al (2007) Efficacy of transcutaneous electrical nerve stimulation in children with overactive bladder. Urology 70(5):980–983 17919697 10.1016/j.urology.2007.06.1109

[16] Hagstroem S, Mahler B, Madsen B et al (2009) Transcutaneous electrical nerve stimulation for refractory daytime urinary urge incontinence. J Urol 182(4):2072–2078 19695629 10.1016/j.juro.2009.05.101

[17] Hoebeke P, De Pooter J, De Caestecker K et al (2009) Solifenacin for therapy resistant overactive bladder. J Urol 182(4):2040–2044 19695608 10.1016/j.juro.2009.05.100

[18] Farhat W, Bagli D, Capolicchio G et al (2000) The dysfunctional voiding scoring system: quantitative standardization of dysfunctional voiding symptoms in children. J Urol 164(3):1011–1015 10958730 10.1016/S0022-5347(05)67239-4

[19] Kim SH, Yoon K, Yoon D et al (2010) An analysis of location of needle entry point and palpated PSIS in S1 nerve root block. The Korean J Pain 23(4):242 21217887 10.3344/kjp.2010.23.4.242 PMC3000620

[20] Barroso U, Carvalho M, Veiga M et al (2015) Urodynamic outcome of parasacral transcutaneous electrical neural stimulation. Int Braz J Urol 41(4):739–743 26401867 10.1590/S1677-5538.IBJU.2014.0303 PMC4757003

[21] Hoffmann A, Sampaio C, Nascimento A et al (2018) Predictors of outcome in children and adolescents with overactive bladder treated with parasacral transcutaneous electrical nerve stimulation. J Pediatr Urol 14(1):54.e1-54.e6 28974365 10.1016/j.jpurol.2017.07.017

[22] de Abreu G, de Souza L, Fonseca M et al (2021) Parasacral transcutaneous electrical nerve stimulation for the treatment of children and adolescents with bladder dysfunction. J Urol 205(6):1785–1791 33525925 10.1097/JU.0000000000001579

[23] Lordêlo P, Soares P, Maciel I et al (2009) Prospective study of transcutaneous parasacral electrical stimulation for overactive bladder in children: long-term results. J Urol 182(6):2900–2904 19846164 10.1016/j.juro.2009.08.058

